# Transcriptome Sequencing Reveals Potential Mechanisms of the Maternal Effect on Egg Diapause Induction of *Locusta migratoria*

**DOI:** 10.3390/ijms20081974

**Published:** 2019-04-23

**Authors:** Kun Hao, Aftab Raza Jarwar, Hidayat Ullah, Xiongbing Tu, Xiangqun Nong, Zehua Zhang

**Affiliations:** State Key Laboratory for Biology of Plant Diseases and Insect Pests, Institute of Plant Protection, Chinese Academy of Agricultural Sciences, Beijing 100193, China; haokun8611@foxmail.com (K.H.); razaaftab282@yahoo.com (A.R.J.); shabkadar@yahoo.com (H.U.); txb1208@163.com (X.T.); xqnong@sina.com (X.N.)

**Keywords:** maternal effect, photoperiodic diapause, RNAi, FOXO pathway, *rai1*

## Abstract

Photoperiod is one of the most important maternal factors with an impact on the offspring diapause induction of *Locusta migratoria*. Previous studies have shown that forkhead box protein O (FOXO) plays an important role in regulating insect diapause, but how photoperiod stimulates maternal migratory locusts to regulate the next generation of egg diapause through the FOXO signaling pathway still needs to be addressed. In this study, the transcriptomes of ovaries and fat bodies of adult locusts under a long and short photoperiod were obtained. Among the total of 137 differentially expressed genes (DEGs) in both ovaries and fat bodies, 71 DEGs involved in FOXO signaling pathways might be closely related to diapause induction. 24 key DEGs were selected and their expression profiles were confirmed to be consistent with the transcriptome results using qRT-PCR. RNA interference was then performed to verify the function of retinoic acid induced protein gene (*rai1*) and *foxo*. Egg diapause rates were significantly increased by RNAi maternal locusts *rai1* gene under short photoperiods. However, the egg diapause rates were significantly decreased by knock down of the *foxo* gene in the maternal locusts under a short photoperiod. In addition, reactive oxygen species (ROS) and superoxide dismutase (SOD) activities were promoted by RNAi *rai1*. We identified the candidate genes related to the FOXO pathway, and verified the diapause regulation function of *rai1* and *foxo* under a short photoperiod only. In the future, the researchers can work in the area to explore other factors and genes that can promote diapause induction under a long photoperiod.

## 1. Introduction

Diapause is an adaptation to seasonality that is widespread across invertebrate taxa which allows them to respond to periodic environmental changes in different developmental stages. Diapause can occur during any stage of development in insects including the egg, larva, pupa and adult stages [1,2,3,4]. However, this diapause induction phase occurs at a genetically predetermined sensitive stage of life, which can be in diapausing individuals or in preceding generations of insects such as silkworm [5,6]. The mosquito *Culex pipiens* can enter a reproductive diapause characterized by an arrest in ovarian development [5]. Similarly, the embryonic diapause termination of *Bombyx mori* is associated with the activation of sorbitol dehydrogenase gene (*SDH*) [7]. A photoperiod signal can regulate the diapause induced by FOXO through an insulin signaling pathway with circadian genes as the input module, meanwhile at the same time, insulin can also regulate the synthesis of juvenile hormone to achieve the diapause process [5,8]. Unlike most insects, diapause induction of locusts is a trans-generational process. Changes due to the environment in the maternal parent could lead to transference of the diapause factor to the offspring eggs [9,10,11]. However, the physiological changes during diapause are largely conserved across species and are hypothesized to be regulated by a conserved suite of genes [12]. The offspring eggs of locusts need to be induced under a low temperature until they cease development in the late anatrepsis stage before the embryo enters diapause [13]. Diapause eggs were found to have relatively strong resistance to cold. The success of egg diapause in winter has a direct effect on the size of locust populations during spring [14]. Maternal effects on transgenerational diapause, especially for the photoperiod, are critical for understanding the locust outbreak dynamics after winter. Hence, *Locusta migratoria* has been used as a model insect to understand the mechanism of insect diapause induction from maternal parents to their offspring.

Our previous studies on diapause induction in migratory locust eggs by transcriptome and proteomic analysis have shown that cellular metabolism in diapause eggs is more active compared to non-diapause eggs where specific enzymes played a role in cryoprotection and provided stored energy for up-regulation in the diapause induction stage [15]. But how the maternal parent is being induced by photoperiod to produce diapause eggs is still unknown. Therefore, it is particularly important to know the molecular mechanism of the maternal effect induced by either a short or long photoperiod. Fat body, a loosely organized tissue in arthropods, has major functions of nutrient storage, hormone synthesis and vitellogenesis besides other vital activities. Vitellogenesis is the process of yolk formation (vitellogenin or egg yolk protein) via nutrients being deposited in the oocyte or female germ cell involved in reproduction of lecithotrophic organisms. In insects, vitellogenesis starts when the fat body stimulates the release of juvenile hormones and produces proteins. Entry into vitellogenesis is an important stage of oogenesis and by forcing females into reproductive diapause, oogenesis can easily be arrested at the pre-vitellogenic stages [16]. Photoperiodic signals are possibly transmitted to eggs by proteins synthesized from fat bodies. Hence, we analyzed the transcriptomes of fat bodies and ovaries of adult migratory locusts induced by long and short photoperiod. The key genes related to diapause induction were obtained and verified for their specific functions by RNAi, and we determined their regulatory relationship with the FOXO signaling pathway. This could be helpful to provide a reference for studying the mechanism of diapause induction in many other insects as well. It also generates insight into the monitoring and managing of pest outbreaks in a specific environment.

## 2. Results

### 2.1. Transcriptomic Analyses

**Figure 1:**
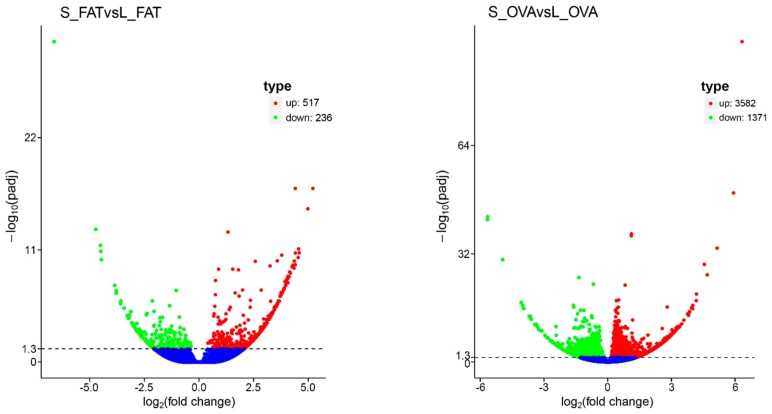
DEGs between long and short photoperiods treatments in *L. migratoria* fat body (left) and ovary (right) samples. DEGSeq (2010) R package (1.10.1) was used to carry out the differential expression analysis in digital gene expression and determining the expression via model based negative binomial distribution. Resulting *p* values were adjusted using the Benjamini and Hochberg’s approach for controlling the false discovery rate. Genes with an adjusted *p*-value of <0.05 explained by DEGSeq were assigned as differentially expressed. The *x*-axis represents the change of gene expression in different groups; whereas the *y*-axis represents the statistical significance of gene expression change. −log10(padj) means −log10 (adjusted *p*-value). The smaller the adjusted *p*-value in −log10(padj), the greater the difference will be (significant). Blue dots in the figure represent the genes with no significant difference; red dots represent the up-regulated genes with significant difference, whereas green dots represent the down-regulated genes with significant difference.

**Figure 2:**
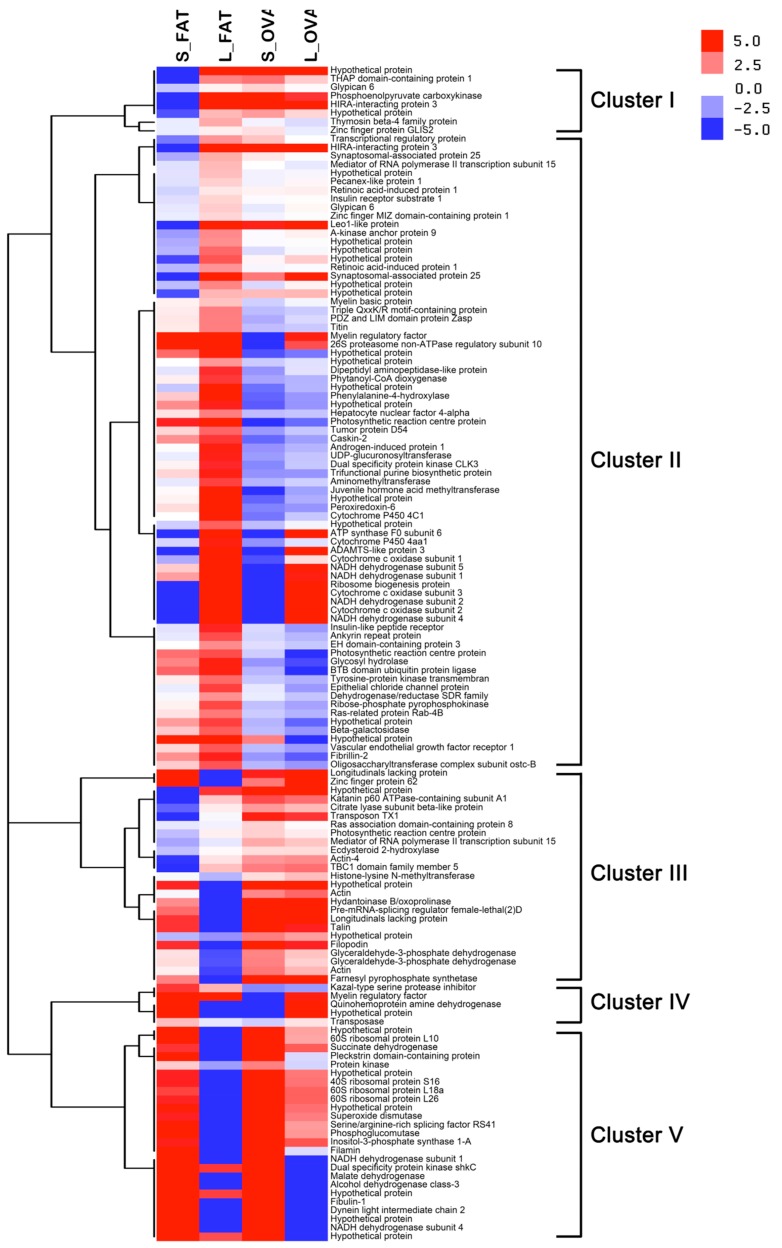
Heatmap of DEGs across the four treatments. Each line in the figure represents a gene, with the columns representing S_FAT, L_FAT, S_OVA and L_OVA. Red indicates relatively high expression and blue indicates relatively low expression. All of the DEGs fall in the range of ±5.0. The spectrum of color from red to blue, indicating that log10 (FPKM + 1) has flow from large to small.

The fat body (FAT) and ovary (OVA) transcriptomes of both long (L) and short (S) photoperiods were sequenced independently. Twelve mRNA libraries were generated from fat body under long (L_FAT) and short photoperiods (S_FAT), and ovary under long (L_OVA) and short photoperiods (S_OVA). Three biological repeats were detected for each group. 75.6–90.7 million clean reads with Q20 > 95% were obtained along with 10.6–13.6 clean bases (Table 1). FAT and OVA transcriptomes were then assembled into 260,779 and 323,527 transcripts individually. Similarly, 102,273 and 132,147 unigenes obtained from FAT and OVA transcriptomes were annotated. To uncover the molecular mechanism underlying these transcriptomic profiles, gene function was annotated based on seven databases including NR, GO, Pfam, SwissProt, KEGG, COG and NT (Table 1) by BLAST (*e*-value < 0.00001). A total of 517 up-regulated and 236 down-regulated transcripts were found in S_FAT versus the L_FAT group. Similarly, for the ovary samples, a total of 3582 up-regulated and 1371 down-regulated transcripts were found in S_OVA versus the L_OVA group (Figure 1). Up-regulated transcripts were ~2.5 times greater than the down-regulated transcripts in both groups, which suggested most genes were induced by a short photoperiod. In addition, the number of DEGs found in the OVA group was ~6 times greater than in the FAT group, which indicated that genes related to photoperiod in ovaries were greater than in fat bodies. Correlation analysis showed that a total of 137 transcripts, including 90 positive and 47 negative correlation DEGs, were differentially expressed in both OVA and FAT groups (Figure S1). To identify the photoperiod induced expression profile in both OVA and FAT groups, the 137 correlated DEGs were subsequently clustered by CLUSTER 3.0 software (Figure 2).

**Cluster I (eight genes)**: photoperiod related genes unique to fat bodies. Total of eight genes were identified in this cluster, including glypican 6, phosphoenolpyrucate carboxykinase, HIRA-interacting protein 3, etc. For this cluster, only DEGs of S_FAT down-regulated while DEG associated with L_FAT and S/L_OVA up-regulated (Figure 2, Table S1). **Cluster II (74 genes)**: negative regulation of photoperiod related genes. We obtained a total of 74 genes where the key negative regulating photoperiod related genes include NADH dehydrogenase, retinoic acid-induced protein 1, insulin receptor substrate 1, etc. For this cluster, expressions of most genes were lower under a short photoperiod than under a long photoperiod in both FAT and OVA (Figure 2, Table S1). **Cluster III (25 genes)**: Cluster III consists of 25 photoperiod related genes that are also unique to fat bodies including actin, longitudinals lacking protein, glyceraldehyde-3-phosphate dehydrogenase, etc. For this cluster, expressions of genes were significantly different in the FAT group as compared to OVA, which was non-significant (Figure 2, Table S1). **Cluster IV (five genes):** This cluster has the minimum number of genes. Photoperiod related genes were inversely expressed in fat bodies and ovaries including kazal-type serine protease inhibitor, myelin regulatory factor, quinohemoprotein amine dehydrogenase, etc. For this cluster, expressions of genes were higher in S_FAT and L_OVA as compared to L_FAT and S_OVA where the expression was lower (Figure 2, Table S1). **Cluster V (25 genes)**: Cluster V has a total of 25 genes where the positive regulation of photoperiod related genes includes ribosomal protein, succinate dehydrogenase, filamin, etc. For this cluster, expression of most genes was higher under a short photoperiod than under a long photoperiod in both FAT and OVA (Figure 2, Table S1).

### 2.2. KEGG Pathways Analysis

To investigate the biological functions, all of the selected DEGs of FAT group were mapped to 193 pathways, while DEGs of OVA group were mapped to 288 pathways by aligning to Kyoto Encyclopedia of Genes and Genomes (KEGG) database. To identify the DEGs related to diapause in FAT and OVA group under the two photoperiods, enrichment analysis was performed for all KEGG pathways. Results showed that ten biological pathways were enriched (*p* < 0.05) in S_FAT vs. L_FAT up-regulated DEGs, including ribosome (ko03010), TCA cycle (ko00020), glycolysis (ko00010), etc., while ten biological pathways were enriched (*p* < 0.05) in S_FAT vs. L_FAT down-regulated DEGs, including oxidative phosphorylation (ko00190), glyoxylate and dicarboxylate metabolism (ko00630), peroxisome (ko04146), etc. Similarly, 19 biological pathways were enriched (*p* < 0.05) in S_OVA vs. L_OVA up-regulated DEGs, including ribosome (ko03010), protein processing in endoplasmic reticulum (ko04141), oocyte meiosis (ko04114), etc., while ten biological pathways were enriched (*p* < 0.05) in S_OVA vs. L_OVA down-regulated DEGs, including the thyroid hormone signaling pathway (ko04919), focal adhesion (ko04510), the longevity regulating pathway (ko04213), etc. (Table 2). To find out some common pathways in both FAT and OVA groups, the correlated DEGs were also annotated to the KEGG database. Oxidative phosphorylation (ko00190), ribosome (ko03010), biosynthesis of secondary metabolites (ko01110), TCA cycle (ko00020) and glycolysis (ko00010) were the top 5 annotated pathways (Table S2). DEGs related to oxidative phosphorylation, including COX1, COX2, COX3, ATPeF0A, ND1, ND2, ND3, ND4, ND5 were down-regulated under a short photoperiod than a long photoperiod in both FAT and OVA groups, while TCA cycle and glycolysis, including MDH1, SDHA, GAPDH, PGM2, were up-regulated. These pathways and genes mentioned above were closely related to the FOXO signaling pathway. In addition, ribosome genes including RP-L10e, RP-L18Ae and RP-L26e were up-regulated under a short photoperiod rather than under a long photoperiod in both FAT and OVA groups (Table S2).

### 2.3. Validations of DEGs Quantitative Real-Time PCR (qRT-PCR)

**Figure 3:**
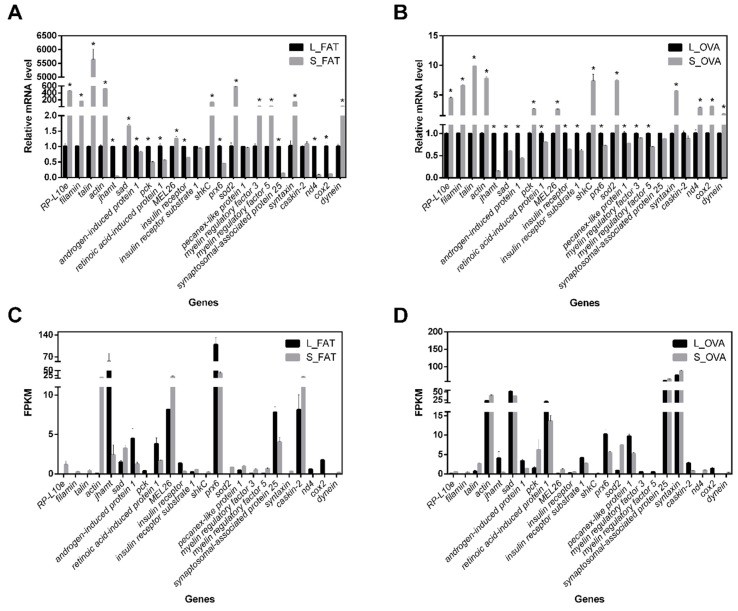
qRT-PCR validations of 24 DEGs in FAT and OVA groups. (**A**) Relative mRNA level of 24 DEGs in FAT group. (**B**) Relative mRNA level of 24 DEGs in OVA group. (**C**) FPKM value of 24 DEGs in FAT group. (**D**) FPKM value of 24 DEGs in OVA group. For each DEG, three technical replications were performed; *ef-1* gene was used as internal control. For each treatment, three replications were performed. Values were reported as mean ± SE. * Indicates an error probability of *p* < 0.05 using Student’s *t*-test.

To confirm the reliability of RNA sequencing data, the mRNA level of 24 DEGs in both FAT and OVA groups including *RP-L10e*, *cox2*, *dual specificity protein kinase* (*shkC*), *retinoic acid-induced protein 1* (*rai1*), etc., were selected randomly for quantitative real-time reverse transcription PCR (qRT-PCR) analysis. The result showed that 43 out of 48 DEGs expression patterns were consistent with RNA sequencing data, though the fold change findings of qRT-PCR and RNA-Seq were not exactly matched (Figure 3). Scatter plots of DEGs mRNA level versus FPKM from RNA-Seq (Figure S2) revealed that our RNA sequencing data are valid as they were significantly correlated (Pearson *R* = 0.7141, *R*^2^ = 0.5099, *F* = 47.860, *p* < 0.0001).

### 2.4. Rai1 and foxo Functions Identified by RNAi

**Figure 4:**
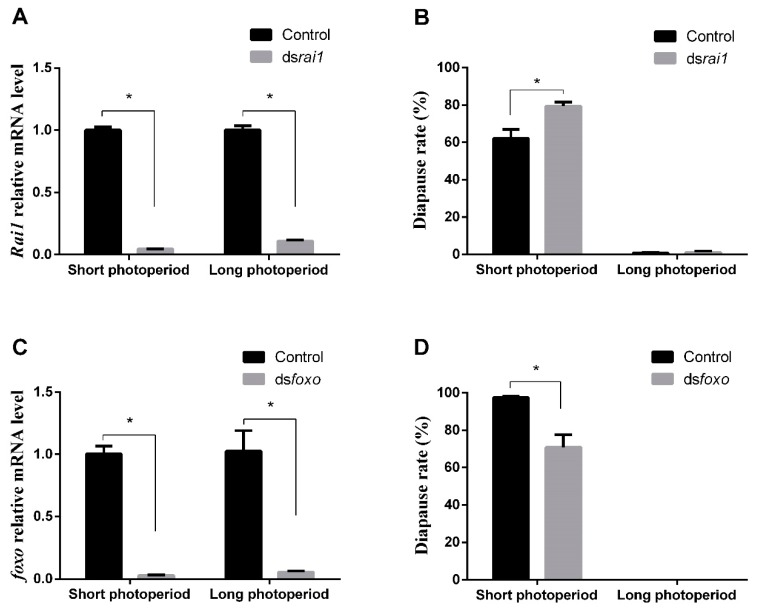
(**A**) RNAi efficiency verified in the whole body of adult females after injecting dsRNA of *rai1* under both long and short photoperiods. (**B**) Diapause rate detected after injecting dsRNA of *rai1* under both long and short photoperiods. (**C**) RNAi efficiency verified in the whole body of adult females after injecting dsRNA of *foxo* under both long and short photoperiods. (**D**) Diapause rate detected after injecting dsRNA of *foxo* under both long and short photoperiods. Numbers of replications were set as three for each treatment. Values were reported as mean ± SE. * Indicates an error probability of *p* < 0.05 using Student’s *t*-test.

Photoperiod’s closely related gene ‘*rai1*’ might play an important part in insect diapause induction. The *rai1* gene was then selected for further identification of the diapause related functions. The *rai1* sequence was initially analyzed by NCBI BLAST. The length of the *rai1* coding sequence was 3243 bp, which could have encoded a protein with 1080 amino acid. However, the percent identity and coverage with *rai1* of *Zootermopsis nevadensis* was 49 and 67, respectively. Similarly, an extended PHD finger found in *rai1* (Ephd_rai1_like domain), in interval of 888–1067 aa of *rai1*. To verify the function of *rai1* on regulating locust diapause, dsRNA of *rai1* was synthesized and subsequently injected into a female adult of *L. migratoria* to RNAi *rai1* under long and short photoperiods, followed by confirming RNAi efficiency via qRT-PCR. It was observed that the mRNA level of *rai1* was significantly (*p* < 0.05) lower in treatment than in control (inject ddH_2_O) under long and short photoperiods, which indicated acceptability of RNAi efficiency of *rai1* (Figure 4A). Under a short photoperiod, average egg diapause rate (DR) was 79.4% in RNAi *rai1* treatment, significantly (*p* = 0.0214) higher than control (62.1%). However, diapause rate had no significant difference between RNAi treatment and control under a long photoperiod. We therefore concluded that RNAi maternal *rai1* could promote offspring diapause under short photoperiod conditions (Figure 4B).

Previous study has suggested that *foxo* was a key gene for diapause induction of the mosquito *Culex pipiens* [8]. To verify the function of *foxo* on inducing locust diapause, ds*foxo* was injected into female adults of *L. migratoria* to knock down the *foxo* gene under long and short photoperiods, followed by confirming RNAi efficiency via qRT-PCR. It was observed that the mRNA level of *foxo* was significantly (*p* < 0.05) lower in treatment than in the control (inject ddH_2_O) under long and short photoperiods, which indicated acceptability of RNAi efficiency of *foxo* (Figure 4C). However, under short photoperiod conditions, the average egg diapause rate (DR) was 97.4% in the negative control treatments, while it was significantly (*p* = 0.0173) lower in the ds*foxo*-treated insects (70.9%). In contrast, the diapause rate was not affected by ds*foxo* treatment under long photoperiod conditions. Thus, we suggest that RNAi maternal *foxo* could inhibit the offspring diapause under short photoperiod conditions (Figure 4D).

### 2.5. SOD and ROS Activity Changes after RNAi rai1

**Figure 5:**
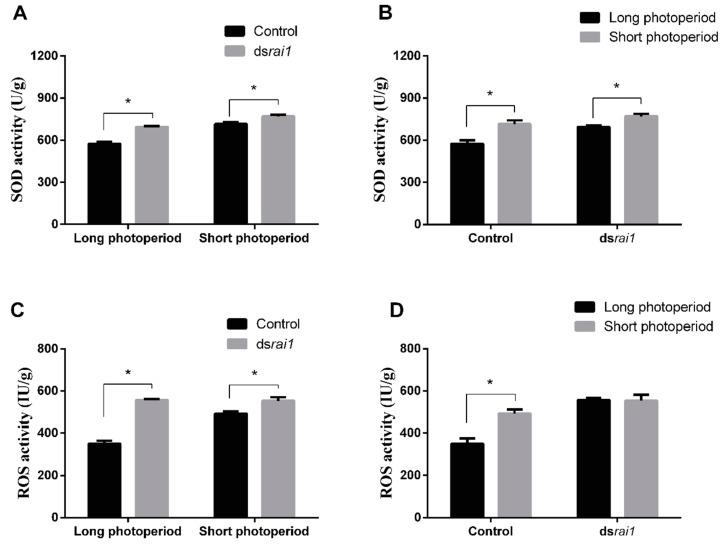
(**A**,**B**) SOD activity detected after injecting dsRNA of *rai1* to locusts under both long and short photoperiods. (**C**,**D**) ROS activity detected after injecting dsRNA of *rai1* to locusts under both long and short photoperiods. For each treatment, three replications were performed. Values were reported as mean ± SE. * Indicates an error probability of *p* < 0.05 using Student’s *t*-test.

Previous study has shown that high ROS level could promote FOXO phosphorylation and subsequently induced diapause. SOD being a typical antioxidant enzyme shows resistance to oxidative damage, diapause and lifespan extension. The effect of ds*rai1* injection on SOD and ROS, and subsequent detection and measurement of SOD and ROS activities in fat body of ds*rai1*-treated and control locusts under both long and short photoperiods were verified. Results showed that SOD activity in RNAi *rai1* treatment was significantly (*p* = 0.0013) higher than control under a long photoperiod. Similarly, SOD activity in RNAi *rai1* treatment was also significantly (*p* = 0.0387) higher than control under a short photoperiod (Figure 5A). Furthermore, SOD activity in a short photoperiod treated sample was significantly higher than that in a long photoperiod treated sample in both ds*rai1* treatment and control (Figure 5B). ROS activities were 557.3 IU/g in RNAi *rai1* treatment, significantly (*p* = 0.0002) higher than control (349.9 IU/g) under a long photoperiod. Meanwhile, ROS activities were 553.8 U/g in RNAi *rai1* treatment, significantly (*p* = 0.0370) higher than control (492.7 U/g) under a short photoperiod (Figure 5C). ROS activities in a short photoperiod treated sample were significantly higher than that in a long photoperiod treated sample in control. However, no significant difference for ROS activities between samples under long and short photoperiods in ds*rai1* treatments were observed (Figure 5D). Results indicated that *rai1* negatively regulated SOD and ROS. SOD and ROS levels were higher under a short photoperiod than a long photoperiod.

## 3. Discussion

Photoperiod is considered as one of the most important environmental factors affecting insect diapause [17,18,19]. The embryonic diapause is transgenerationally induced and controlled by the maternal parent in *Bombyx mori* [20]. Our previous studies showed that female adults of *L. migratoria* could also be induced by a short photoperiod and subsequently produce diapause eggs [13,21]. Here, fat bodies and ovaries of locust under long and short photoperiods were used for transcriptomic analysis. The correlated DEGs between OVA and FAT group were validated by qRT-PCR. In this study, we focused on finding the important maternal DEGs, which respond to diapause induction signals (photoperiod), and contribute to a better understanding of the molecular mechanisms underlying insect diapause induction.

### 3.1. DEGs and Pathways Related to FOXO Signaling Pathway

Photoperiods are important cues for seasonal adaptations in insects. Although egg diapause is one of the seasonal adaptations, a photoperiod change is likely to affect many aspects of physiological status. FOXO signaling is involved in a wide variety of insect phenomena. Thus, FOXO signaling genes might be stimulated by photoperiods. FOXO’s role in diapause induction has been noted in some other invertebrates, including nematodes [22] and mosquitoes [8]. FOXO activity is inhibited by growth factors that ultimately affect insulin signaling pathways, stimulated by nutrient depletion and a plethora of ROS-induced post-translational modifications [23]. It has been observed that the insulin signaling and FOXO, a downstream molecule in the insulin signaling pathway, mediate the diapause response in *Culex pipiens* [8]. In this study, DEGs were grouped into five clusters to find out their functions related to diapause induction under photoperiod regulation. These DEGs were also mapped to KEGG pathways to understand their role in up or down regulation. Results indicated that many of these DEGs (INSR, IRS, MnSOD, EGFR, etc.) and pathways (ko00010, ko00020, ko00190) were involved in the FOXO signaling pathway (Table S3).

qRT-PCR results showed that relative expression of *INSR* and *IRS* was significantly lower in samples under a short photoperiod than in samples under a long photoperiod. Under a short photoperiod, down-regulation of *INSR* and *IRS* expression suggest that the insulin signaling pathway was possibly inhibited and as a result of that the FOXO was more active in locusts under a short photoperiod. In addition, *MnSOD* has already been identified as an important target downstream gene of FOXO in mice and nematodes [24,25]. Expression of *MnSOD* (*sod2*) and SOD activity was significantly higher in samples under a short photoperiod than in samples under a long photoperiod. The up-regulation of *MnSOD* expression and SOD activity also implied that FOXO was activated during the diapause induction stage of *L. migratoria*. RNAi results demonstrated that knocking down the *foxo* gene of maternal locusts decreased the diapause incidence of offspring under a short photoperiod. This result is consistent with the previous findings that FOXO is active under short photoperiod conditions in mosquitoes [8], and indicates that FOXO could likewise be important in the photoperiod-induced diapause process of *L. migratoria*.

**Figure 6:**
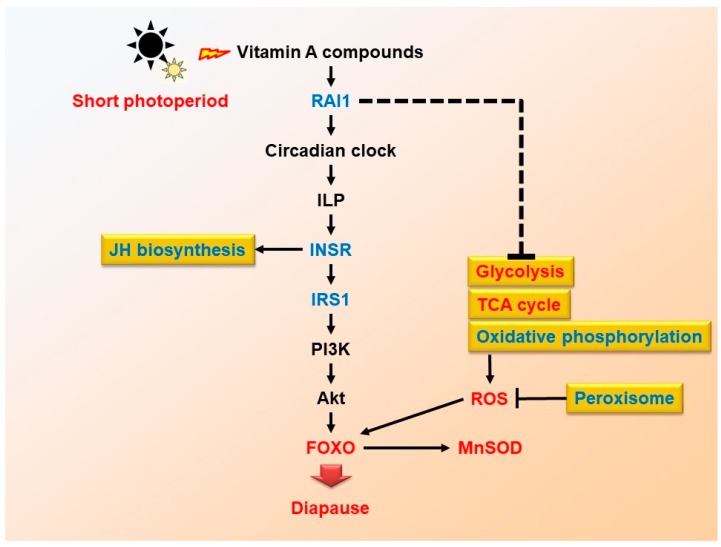
Potential pathway of maternal effect on egg diapause induction of *L. migratoria*. The ‘blue’ mean ‘down-regulated’, ‘red’ means ‘up-regulated’ whereas ‘black’ means ‘not confirmed in our experiment’.

The insect hormone biosynthesis pathway (ko00981) was also very important in diapause induction according to the transcriptome. Transcriptome analysis suggested up-regulation of ecdysteroid biosynthesis in a diapause-inducing group of *Bombyx mori* [20]. Furthermore, Juvenile hormone and ecdysteroids were involved in egg diapause of *L. migratoria* [26,27]. Gene such as *fpps* has a key role in synthesis of terpenoid backbone [28,29]. Similarly, *sad* has been identified to be vital for ecdysone synthesis in *Drosophila melanogaster* [30]. In our study the transcriptome analysis revealed the up-regulation of *fpps* and *sad* in a diapausing-induced group of *L. migratoria* as compared to a non-diapausing group. Meanwhile, *JHamt* is known for its critical and closely related role in JH biosynthesis, which is associated with insulin signaling pathways [31,32]. *JHamt* is down-regulated in locusts under a short photoperiod, which means JH biosynthesis must have decreased. Studies have also revealed that the insulin promotes JH biosynthesis [33,34,35]. The decrease in JH synthesis also indicated that the insulin signaling pathway has been inhibited and subsequently indicated the activation of FOXO (Figure 6).

Another important feature of the FOXO signaling pathway (ko04068) was ROS. DEGs involved in glycolysis and TCA cycle, including *pgm*, *gapdh*, *adh*, *sdh1*, *mdh1*, were up-regulated in samples under a short photoperiod. In contrast, DEGs involved in oxidative phosphorylation and peroxisome, including *prx6*, *nd4*, *cox2*, were down-regulated in fat body samples under a short photoperiod. However, both qRT-PCR and transcriptomic results showed that *nd4* was up-regulated in ovary samples under a short photoperiod. This has clearly indicated almost all of the DEGs involved in glycolysis and TCA cycle induced ROS. ROS was reported as central regulator in inducing the diapause in *Helicoverpa armigera* [36], while also keeping the FOXO active. ROS activities detection also showed that ROS level is higher in samples under a short photoperiod than a long photoperiod (Figure 5D). This suggested that the ROS must keep a relatively higher level in maternal locust for diapause induction (Figure 6).

Interestingly, DEGs of ribosome were all up-regulated in samples under a short photoperiod. This indicates that ample supplies of ribosomal bodies are needed during diapause induction. These ribosomal reserves may even be transmitted directly to the eggs. In addition, there are other pathways associated with diapause induction, such as focal adhesion including *β-actin*, *flamin*, *tilin*, etc.; neurogenesis, including *pecanex*, *mrf* and *mbp*; synaptic vesicle cycle, including *SNAP25* and *syntaxin* (Table S3).

### 3.2. Maternal rai1 Regulates Locust Diapause

Both transcriptome analysis and RT-PCR results showed that mRNA level of *rai1* was lower in samples under a short photoperiod than that of under a long photoperiod. This indicated that *rai1* expression was repressed by short photoperiod. In addition, egg diapause could have induced under short photoperiod. This suggested that the diapause induction in maternal locust was possibly negatively regulated by *rai1*. To confirm our hypothesis, RNAi was performed to identify the diapause regulation function of *rai1*. The result showed that the diapause incidence in *L. migratoria* increased after knocking down of the *rai1* gene (Figure 4B). The RNAi result was consistent with the *rai1* expression profiles obtained through transcriptome analysis and RT-PCR. Hence, we concluded that the offspring diapause could be inhibited by a short photoperiod through repressing maternal *rai1* expression. For the first time in 1997, *rai1* was reported as a retinoic acid induced gene of acute promyelocytic leukemia cell [37]. Vitamin A is a group of unsaturated nutritional organic compounds that includes retinol, retinal, retinoic acid, and several provitamin A carotenoids. These retinoid compositions were found in insect eyes [38]. Photoperiodic induction of diapause in *Apanteles glomeratus* and *Amblyseius potentillae* has also been known to be Vitamin A dependent [39,40]. Previous investigation into the role of *rai1* revealed that it has regulated circadian genes including *per*, *cry1*, etc. in mice. [41]. In addition, circadian genes had an input module to transmit a light signal to the insulin signaling pathway and finally lead *C. pipiens* diapause [33]. Circadian clock genes along with carbohydrate, lipid and energy metabolism are the known vital drivers for activating the environmental signals in summer diapause of *Delai antiqua* [42]. Lipid metabolism can be seen distinctly in later phase of diapause however, understanding the difference in dormancy and diapausing, the lipid metabolism along with hormonal control clearly explained *Aedes albopictus* (Asian tiger mosquito) development through transcriptome analysis [43]. Due to the complex nature of diapause, a number of genes and processes regulate the diapausing in *Drosophila montana* including metabolism and fatty acid biosynthesis, which assist diapausing females to survive overwintering [44]. So, we speculate that photoperiodic signals are transmitted to maternal *rai1* via retinoic acid and eventually to the circadian genes and downstream insulin signaling pathway, which leads to offspring’s egg diapause (Figure 6) which supports the previously reported findings in *Delia antiqua* [42]. Similarly, a high ROS level induced pupal diapause of *Helicoverpa armigera* and extended the lifespan of insects [45,46]. *Sod-2*, a downstream gene of FOXO, was dramatically higher in diapausing females than in non-diapausing females of mosquito *C. pipiens* [47]. Results showed that ROS and SOD activities were higher in ds*rai1* treatment than in control. The up-regulation of SOD activity implied that FOXO is being activated after RNAi *rai1*. These results suggested that RAI1 might inhibit diapause induction by repressing ROS and FOXO activities. On the other hand, retinoic acid has also been found to be critical for neurogenesis of *L. migratoria* [48]. Results in the present study suggested that other factors might inhibit induction of *L. migratoria* diapause under a long photoperiod. Only knocking down ds*rai1* might not enough to cut-off the insulin signaling and subsequently regulates the FOXO activity under a long photoperiod. This could be the main reason why egg diapause enhancement by knocking down *rai1* was confirmed only under a short photoperiod, whereas it was not observed under a long photoperiod.

Egg diapause enhancement through ds*rai1* treatments was confirmed under short photoperiod. Due to the complex nature of diapause regulation, the *rai1* can regulate diapause; however, this may not be the final decisive role of *rai1*. Results showed that knocking down *rai1* could only increase the diapause rate under short photoperiod; although, a 100% increase in diapause is not determined by the study undertaken. In mosquitoes, the phosphorylated FOXO was blocked to translocate into the nucleus by insulin signaling pathway under long-day photoperiod [8]. We presumed that insulin signaling pathway strongly arrested FOXO activity. Only knocking down ds*rai1* might not enough to cut-off the insulin signaling and subsequently regulates the FOXO activity under a long photoperiod. This could be the main reason why knocking down *rai1* cannot promote egg diapause of *L. migratoria* under a long photoperiod.

In the present study, dozens of DEGs were obtained in different tissues of maternal *L. migratoria* induced by different photoperiods. Most of these DEGs, including *INSR*, *IRS*, *MnSOD*, *EGFR*, were closely related to the FOXO signaling pathway. Among these DEGs, the *rai1* was selected and confirmed to be involved in diapause induction of maternal locust under a short photoperiod. Because of the special relationship between retinoic acid and light, we are of the opinion that the photoperiod was likely to affect the FOXO signaling pathway through the maternal *rai1*, and ultimately established environmental signals, transmitted to the next generation to induce locust egg diapause. Future researchers can work in the area to explore other factors and genes that can promote diapause induction under a long photoperiod, and can highlight the specific mechanism of related DEGs regulating diapause through the FOXO signaling pathway.

## 4. Materials and Methods

### 4.1. Insect Rearing and Tissue Collection

The *L. migratoria* L. colony used in this study was originally collected from the field at Tianjin, China (38°49′ N, 117°18′ E) in November 2007 and was maintained by the State Key Laboratory for Biology of Plant Diseases and Insect Pests, Institute of Plant Protection, Chines Academy of Agricultural Sciences. Third instar locusts were collected from the rearing cages and transferred to 20 cm × 20 cm × 28 cm mesh cages in artificial climate chambers (PRX-250B-30, Haishu Saifu Experimental Instrument Factory, Ningbo, China) where the insects were raised to adults at either 27 °C and 60% RH under a long photoperiod of 16:8 L:D to produce non-diapause eggs, and at 27 °C, 60% RH under a short photoperiod of 10:14 L:D to produce diapause eggs. When *L. migratoria* matured after 72 h under both long and short photoperiods, ovaries and fat bodies were separately collected from female individuals. Ovaries were dissected at the stage of developmental phase-II. In phase II, during the process of vitellogenesis, the color of the ovaries turned to light yellow from white, had an ovary size of around 10 mm × 5 mm (length × width), whereas the fallopian tube shifted from thin to round. Tissues were dissected into the ice-cold RNase-free saline solution. There were four tissues and three replicates in each tissue. All of the samples were immediately frozen by liquid nitrogen and then kept at −80 °C until RNA extraction. The *L. migratoria* L. colony used in this study has been maintained by the State Key Laboratory for Biology of Plant Diseases and Insect Pests, Institute of Plant Protection, Chines Academy of Agricultural Sciences. The locusts are common agricultural pests and are not included in the “List of Endangered and Protected Animals in China”.

### 4.2. RNA Extraction and RNA-Seq

A total of twelve *L. migratoria* tissues were used whereas RNA extraction, library construction and sequencing were carried out as described in previously reported study [21]. Total RNA was isolated from frozen medullar tissue by using the RNA plant mini kit with column DNase digestion (Qiagen, Hilden, Germany) following the manufacturer’s instructions. RNA concentration was then measured using Qubit RNA Assay Kit in Qubit 2.0 Flurometer (Life Technologies, Carlsbad, CA, USA). Additionally, RNA integrity was assessed using the RNA Nano 6000 Assay Kit of the Bioanalyzer 2100 system (Agilent Technologies, Santa Clara, CA, USA). All of the 12 samples with RNA integrity numbers (RIN) above 8 were used for construction of the libraries. Sequencing libraries were generated using NEBNext Ultra™ RNA Library Prep Kit for Illumina (NEB, Illumina, San Diego, CA, USA) following manufacturer’s recommendations, whereas index codes were added to attribute sequences to each sample. Finally, PCR products were purified (AMPure XP system, Beckman Coulter Life Sciences, Indianapolis, IA, USA) and library quality was assessed on the Agilent Bioanalyzer 2100 system. The clustering of the index-coded samples was performed on a cBot Cluster Generation System using TruSeq PE Cluster Kit v3-cBot-HS (Illumia) according to the manufacturer’s instructions. After cluster generation, the library preparations were sequenced on an Illumina Hiseq 2500 platform and 125 bp paired-end reads were generated.

### 4.3. Sequence Assembly, Annotation and DEGs Analysis

Sequencing data of ovary and fat body samples were analyzed independently. Raw data were processed into clean data by eliminating adapters, poly-N and inferior quality reads. Transcriptome assembly was achieved based on the clean data, using Trinity with min_kmer_cov set to default value of 2, whereas all other parameters were set-up to default [49]. Gene function was annotated based on the following seven databases: Nr (NCBI non-redundant protein sequences), Nt (NCBI non-redundant nucleotide sequences), Pfam (Protein family), KOG/COG (Clusters of Orthologous Groups of proteins), Swiss-Prot (a manually annotated and reviewed protein sequence database), KO (KEGG Ortholog database) and GO (Gene Ontology). Data for each sequenced library was analyzed using BLAST with a cut-off *e*-value of 10^−5^. Prior to differential gene expression analysis, for each sequenced library, the read counts were then adjusted by the edge R program package through one scaling normalized factor. Differential expression analysis of two samples were performed using the DEGseq (2010) R package (1.10.1) [50]. The *p*-value was adjusted using *q*-value [51] whereas the *q*-value < 0.05 and |log2 (fold change)| > 1 was specified as the threshold for significantly differential expression.

### 4.4. cDNA Synthesis and qRT-PCR

Total RNAs of L_FAT (L_FAT1, L_FAT2 and L_FAT3 mixed together), S_FAT (S_FAT1, S_FAT2 and S_FAT3 mixed together), L_OVA (L_OVA1, L_OVA2 and L_OVA3 mixed together) and S_OVA (S_OVA1, S_OVA2 and S_OVA3 mixed together) were respectively used to move along with further research. cDNA was synthesized from the four mixed RNA samples using M-MLV reverse transcriptase and recombinant RNase inhibitor (Takara, Beijing, China). The expression levels of 24 DEGs in all four tissues were determined by qRT-PCR using SYBR Premix Ex Taq kit (Takara) as per the manufacturer’s instructions in an ABI 7500 real-time PCR system (Applied Biosystems, Foster City, CA, USA). qRT-PCR was performed as per following conditions: 95 °C for 10 min; 40 cycles of 95 °C for 15 s, 60 °C for 45 s. Gene expression was quantified using 2^−ΔΔ*C*t^ method [52], with elongation factor 1 (*ef-1*) as the internal control for normalization of data. The specific primers used for qRT-PCR are listed in Table S4.

### 4.5. RNA Interference

The dsRNA was generated by in vitro transcription using the T7 RiboMAX system (Promega, Fitchburg, WI, USA) as per prescribed manufacturer’s protocol. Templates for in vitro transcription reactions were prepared by PCR amplification from plasmid DNA of the cDNA clone of *rai1* and *foxo* using the primer pairs with T7 polymerase promoter sequence at 5′-end (Table S4). The length of ds*rai1* was 583 bp, whereas the length of ds*foxo* was 194 bp. A Total of 5 μL of dsRNAs (2 μg/μL) for the target gene (*rai1* or *foxo*), water as controls were used to inject into the ventral part between 2nd and 3rd abdominal segments of the female adults within 72 h after molting. For each gene, 75 female adults were injected and divided into three groups. The effects of RNAi on the mRNA levels were investigated by qRT-PCR at 48 h after injection. To keep track of the transcript levels of *rai1* and *foxo*, total RNA was extracted from entire bodies of locusts. For each target gene, three individuals from each group were used for RNA extraction.

### 4.6. Diapause Rate Detection

Locusts were placed in new mesh cages (20 cm × 20 cm × 28 cm) and provided with bouquets of greenhouse grown wheat after applying the above-mentioned treatments. Meanwhile, 25 adult males were introduced to each replicate. The floor of the cages was covered in a 2 cm layer of sieved sterile sand with the cages maintained until eggs laying by the locusts. Once oviposition was observed, eggs were collected at an interval of 48 h for 10 days using a camel paint brush and transferred into plastic Petri dishes (90 mm × 50 mm), where the eggs were incubated on vermiculite, before shifting to 27 °C and 60% RH to slow down the development. Around 150 eggs were obtained from 2–3 pods, which were then used in each experimental replication. Eggs were kept under 27 °C for 30 days until eclosion of 1st instar nymphs ceased (H1). To account for non-viable eggs, all remaining un-eclosed eggs were kept at 4 °C for 60 days to receive ample time to break the diapause, afterwards they were incubated at 27 °C for 30 days and for any further 1st instar emergence recording (H2). The diapause rate (DR) was calculated as: DR (%) = H2/(H1 + H2).

### 4.7. SOD and ROS Activity Detection

Rapid ELISA-based quantification was used to monitor and measure the SOD and ROS activities in the fat bodies of *L. migtatoria* using the specified manufacturer instructions for catalogue SU-B97128; SU-B97124; SU-B97136; SU-B97141 (Collodi Biotechnology Co., Ltd., Quanzhou, China). The fat body samples were homogenized in a 1 mL PBS, and the resulting suspension was subjected to ultra-sonication to further disrupt the cell membranes. After homogenates were centrifuged for 15 min at 2348× *g* (5000 rpm), the supernatants were collected and were stored at −20 °C until used for further analysis. All of the required reagents and samples were prepared and were properly maintained at room temperature (18–25 °C) for 30 min prior initiating further assay procedure (Table S5). We set-up standard wells, sample wells and blank (control) wells. We then added 50 μL standard to each standard well, 50 μL of sample to each sample well and 50 μL of sample diluent to each blank/control well. A 100 μL amount of HRP-conjugate reagent was added to each well and covered with an adhesive strip and incubated for 60 min at 37 °C. The Micro titer plates were rinsed using Wash Buffer (1×) 4-times followed by adding gently mixed Chromogen Solution A (50 μL) and Chromogen Solution B (50 μL) to each well in succession, protected from light and incubated for 15 min at 37°C. Finally, 50 μL Stop Solution was added to each well. During the process, well color changed from blue to yellow, showed a proper sign and confirmation of uniformity. Colorless or green color is usually a sign of no uniformity. In such case, the plate was then gently tapped to ensure thorough mixing. The Optical Density (OD) at 450 nm was read using a Micro Elisa Strip plate reader (Multiskan™ FC 51119000, Thermo Fisher Scientific Inc., Waltham, MA, USA) within 15 min of adding the Stop Solution. Standard curves of SOD and ROS were constructed respectively and calculated accordingly to quantify the amount of SOD and ROS of each sample.

### 4.8. Statistical Analysis

The differences between treatments were compared by Student’s *t*-test. Differences were considered significant at *p* < 0.05. Values were reported as mean ± SE. Data were analyzed using the SPSS software (version 15.0; SPSS Inc., Chicago, IL, USA) and GraphPad Prism software (version 6.01; GraphPad Software Inc., San Diego, CA, USA).

### 4.9. Availability of Data and Materials

All RNA-seq.fastq files are available from the NCBI Sequence Read Archive database (accession number SRR5762723, SRR5762724, SRR5762725, SRR5762726, SRR5762727, SRR5762728, SRR5754272, SRR5754273, SRR5754274, SRR5754275, SRR5754276, SRR5754277, SRR5754278). All other data is contained in the manuscript and in the Supplementary Materials.

## Figures and Tables

**Table 1:** Summary of RNA-seq metrics from *L. migratoria* transcriptomes for ovaries and fat bodies under both long and short photoperiods.

Sample	Clean Reads	Clean Bases (G)	Q20 (%)	Number of Transcripts	Number of Unigenes
L_FAT1	78872568	11.83	95.61	260,779	102,273
L_FAT2	79227948	11.88	95.62		
L_FAT3	88069778	13.21	95.86		
S_FAT1	77266106	11.59	96.06		
S_FAT2	77647066	11.65	96.78		
S_FAT3	76502530	11.48	95.71		
L_OVA1	80389650	12.06	95.58	323,527	132,147
L_OVA2	90730566	13.61	95.84		
L_OVA3	71022586	10.65	95.30		
S_OVA1	75628016	11.34	95.92		
S_OVA2	88328188	13.25	96.35		
S_OVA3	76316318	11.45	94.97		

**Table 2:** DEGs of different groups enriched KEGG pathways.

Group	Num.	Term	ID	*p*-Value
S_FAT vs. L_FAT up DEGs	1	Ribosome	ko03010	1.62 × 10^−9^
	2	Citrate cycle (TCA cycle)	ko00020	0.003078
	3	Regulation of actin cytoskeleton	ko04810	0.012542
	4	Glyoxylate and dicarboxylate metabolism	ko00630	0.015929
	5	Glycosphingolipid biosynthesis	ko00603	0.019268
	6	Protein processing in endoplasmic reticulum	ko04141	0.02064
	7	Endocytosis	ko04144	0.021813
	8	Glycolysis / Gluconeogenesis	ko00010	0.022633
	9	Cysteine and methionine metabolism	ko00270	0.029743
	10	Fc γ R-mediated phagocytosis	ko04666	0.029743
S_FAT vs. L_FAT down DEGs	1	Glycine, serine and threonine metabolism	ko00260	2.21 × 10^−5^
	2	Oxidative phosphorylation	ko00190	0.000119
	3	Glyoxylate and dicarboxylate metabolism	ko00630	0.000133
	4	Pentose phosphate pathway	ko00030	0.000141
	5	Phenylalanine, tyrosine and tryptophan biosynthesis	ko00400	0.002921
	6	Fructose and mannose metabolism	ko00051	0.006848
	7	Phenylalanine metabolism	ko00360	0.013337
	8	Alanine, aspartate and glutamate metabolism	ko00250	0.019824
	9	Peroxisome	ko04146	0.036338
	10	Arginine biosynthesis	ko00220	0.04393
S_OVA vs. L_OVA up DEGs	1	Protein processing in endoplasmic reticulum	ko04141	3.86 × 10^−11^
	2	Proteasome	ko03050	7.15 × 10^−7^
	3	Spliceosome	ko03040	8.36 × 10^−5^
	4	Cell cycle	ko04110	0.000107
	5	Protein export	ko03060	0.000334
	6	RNA transport	ko03013	0.000422
	7	Antigen processing and presentation	ko04612	0.000672
	8	Ubiquitin mediated proteolysis	ko04120	0.001973
	9	Pyrimidine metabolism	ko00240	0.009155
	10	NOD-like receptor signaling pathway	ko04621	0.010726
	11	DNA replication	ko03030	0.013664
	12	Toll-like receptor signaling pathway	ko04620	0.019418
	13	RNA degradation	ko03018	0.021142
	14	Oocyte meiosis	ko04114	0.021603
	15	Ribosome	ko03010	0.027409
	16	Cytosolic DNA-sensing pathway	ko04623	0.033442
	17	Progesterone-mediated oocyte maturation	ko04914	0.034639
	18	NF-kappa B signaling pathway	ko04064	0.042894
	19	N-Glycan biosynthesis	ko00510	0.048725
S_OVA vs. L_OVA down DEGs	1	Thyroid hormone signaling pathway	ko04919	0.000812
	2	Focal adhesion	ko04510	0.000986
	3	Hippo signaling pathway - fly	ko04391	0.00245
	4	Notch signaling pathway	ko04330	0.009375
	5	Tight junction	ko04530	0.01335
	6	Dorso-ventral axis formation	ko04320	0.018136
	7	Cardiac muscle contraction	ko04260	0.021591
	8	ECM-receptor interaction	ko04512	0.027292
	9	Longevity regulating pathway	ko04213	0.034335
	10	cGMP-PKG signaling pathway	ko04022	0.042573

## Supplementary Materials

Supplementary materials can be found at https://www.mdpi.com/1422-0067/20/8/1974/s1.

## Abbreviations

FAT	fat body
OVA	ovary
L	long photoperiod
S	short photperiod
DEGs	differentially expressed genes
vs	versus
FDR	false discovery rate
BLAST	basic local alignment search tool
GO	Gene Ontology
KEGG	Kyoto Encyclopedia of Genes and Genomes
KO	KEGG Ortholog database
KOG/COG	Clusters of Orthologous Groups of proteins
NR	NCBI non-redundant protein sequences
NT	NCBI nucleotide sequences
Pfam	protein families
Swiss-Prot	a manually annotated and reviewed protein sequence database
TCA cycle	tricarboxylic acid cycle
*fpps*	farnesyl diphosphate synthase
*JHamt*	juvenile hormone acid *O*-methyltransferase
SOD	superoxide dismutase
PGM	phosphoglucomutase
GAPDH	glyceraldehyde 3-phosphate dehydrogenase
ADH	alcohol dehydrogenase
MDH	malate dehydrogenase
INSR	insulin receptor
IRS	insulin receptor substrate
EGFR	endothelial growth factor receptor
COX	cytochrome c oxidase
ND	NADH dehydrogenase
DR	diapause rate
*foxo*	*foxo* gene
FOXO	FOXO protein
