# Supplementary material for: Transcriptome Sequencing Reveals Potential Mechanisms of the Maternal Effect on Egg Diapause Induction of Locusta migratoria
Source: Int J Mol Sci. 2019 Apr 23;20(8):1974. doi: 10.3390/ijms20081974 (PMC6514766; doi:10.3390/ijms20081974)
Supplement: Supplementary file 1 [file ijms-20-01974-s001.zip › Supplementary files/Captions for figures and tables in supplementary file.pdf]

Figure S1. DEGs correlation analysis between S_OVA vs L_OVA group and S_FAT vs L_FAT group.

Figure S2. Scatter plots of gene's total transcript level measured by real-time PCR (log2-transformed) versus estimation from RNA-Seq (log2-transformed FPKM) for *Locusta migratoria* transcriptome.

Table S1. Original data for making cluster diagram

Table S2. KEGG annotation of the 137 correlation DEGs

Table S3. Gene annotation of the 137 correlation DEGs

Table S4. Primers designed for qRT-PCR and RNAi

Table S5. Composition of ELISA reagents for protein phosphorylation analysis
