# Supplementary figures and images for: Transcriptome Sequencing Reveals Potential Mechanisms of the Maternal Effect on Egg Diapause Induction of Locusta migratoria
Source: Int J Mol Sci. 2019 Apr 23;20(8):1974. doi: 10.3390/ijms20081974 (PMC6514766; doi:10.3390/ijms20081974)

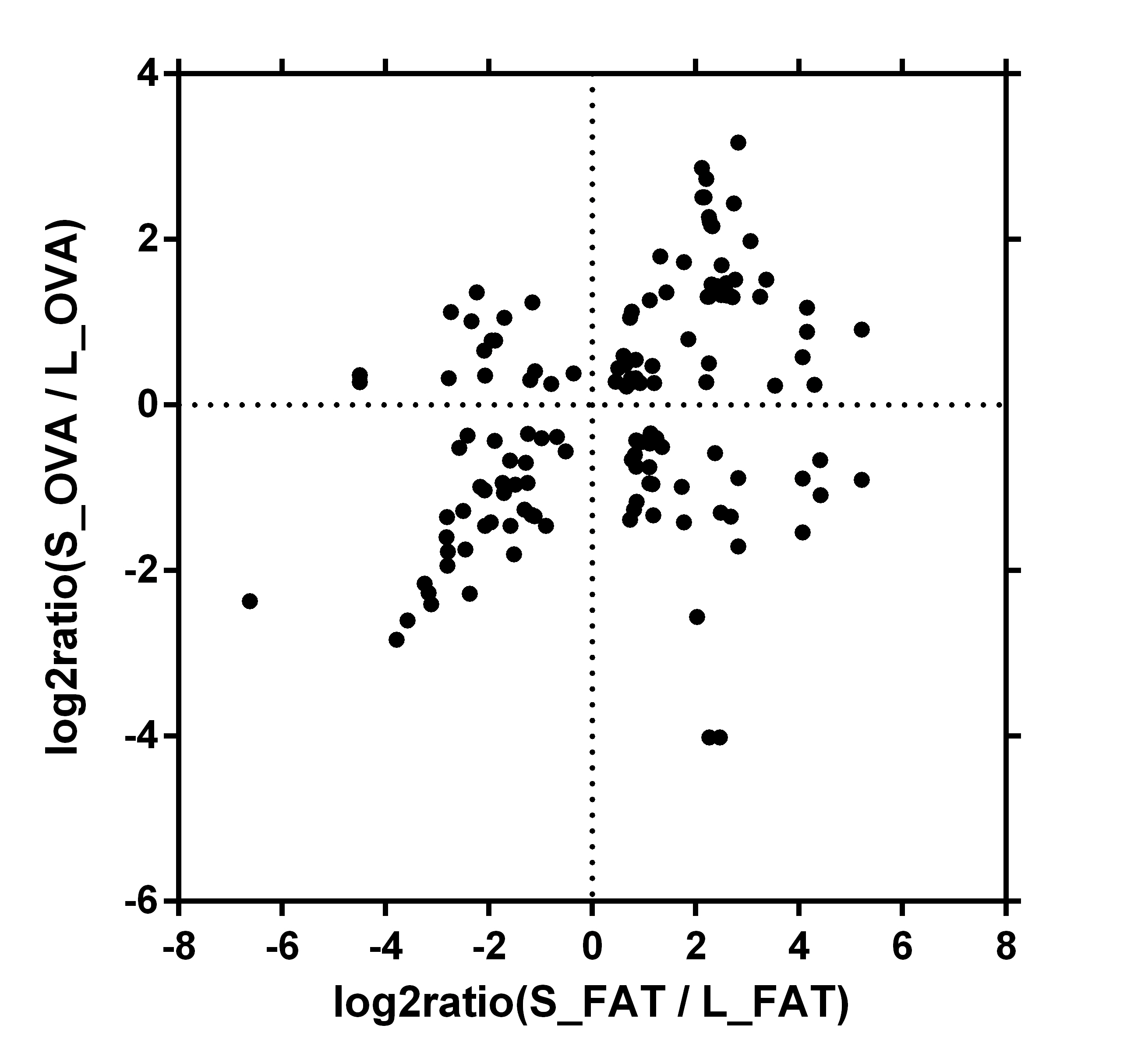

Supplement: Supplementary file 1 [file ijms-20-01974-s001.zip › Supplementary files/Figure S1.jpg]

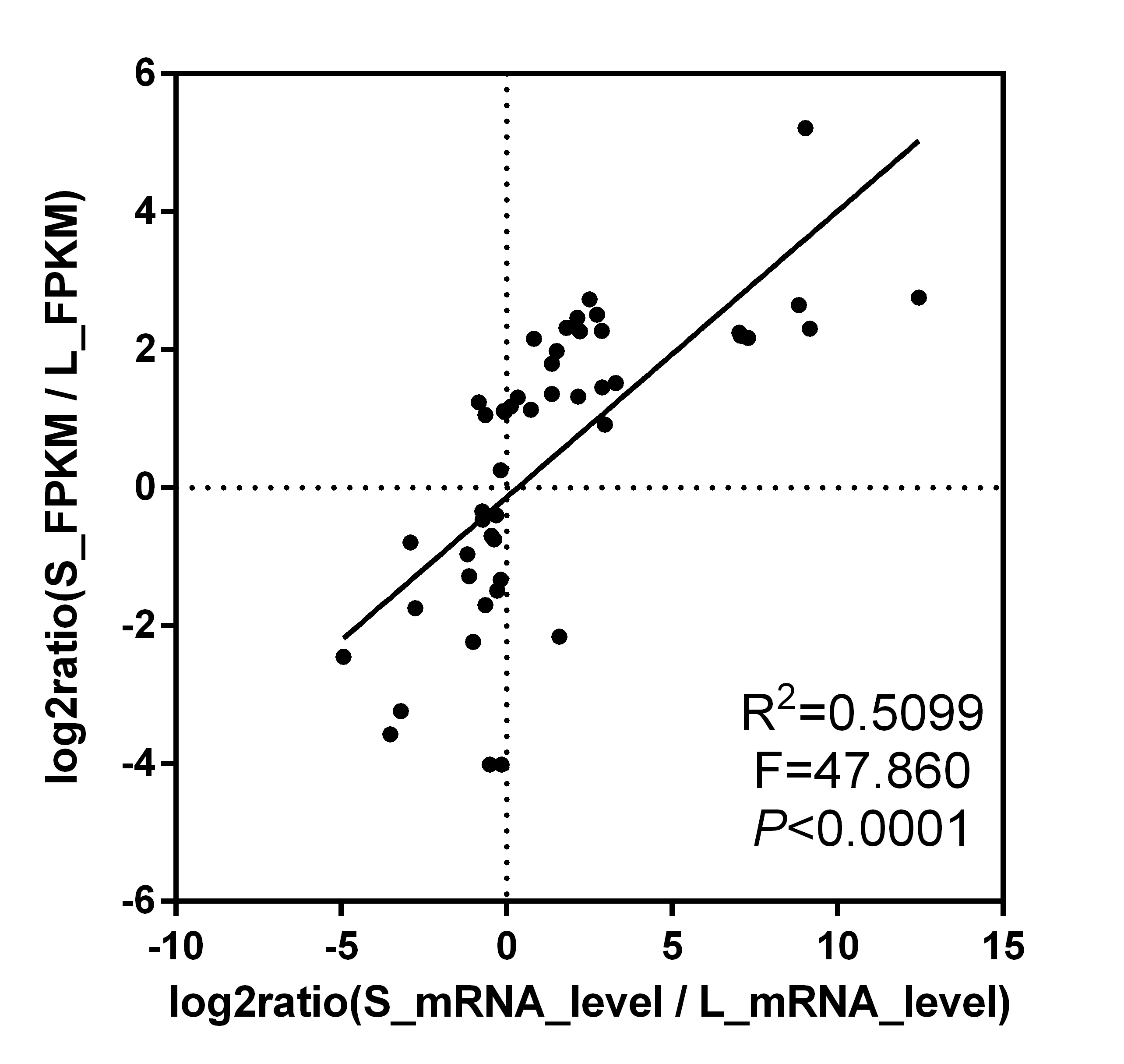

Supplement: Supplementary file 1 [file ijms-20-01974-s001.zip › Supplementary files/Figure S2.jpg]
